# Editorial: The role of technology and social media in sport

**DOI:** 10.3389/fspor.2025.1750115

**Published:** 2025-12-10

**Authors:** Shir Levy, Christopher Barry, Jolita Vveinhardt

**Affiliations:** 1 Department of Psychology, Washington State University, Pullman, WA, United States; 2 Institute of Sport Science and Innovations, Lithuanian Sports University, Kaunas, Lithuania

**Keywords:** social media engagement, athlete well-being, social comparison, sports psychology, fan engagement

In recent years, various technological innovations have transformed the sports world. Specifically, advancements, such as publicly available statistics and performance data, interactions with fans across online platforms, and social media use, have prompted significant changes in the daily lives of athletes and sports teams worldwide. Among these trends, social media platforms play a particularly significant role. Social media provides unique opportunities for athletes and teams, and these applications are often used to enhance athlete status and careers (e.g., through sponsorship opportunities; community building). However, they may also increase psychological pressure (e.g., through social comparison, pressure to perform, or negative online interactions), adding a new layer of stress to an already demanding lifestyle.

Previous research has demonstrated the various influences of social media exposure on people's well-being, perceptions, and social relationships (1–3). However, there has been limited empirical research dedicated to understanding the role of social media in the sports industry. Although the topic of media exposure and its consequences is relevant across populations, athletes can be considered a unique population with distinct circumstances. Specifically, athletes, who already operate in performance-oriented environments, may be more susceptible to the influence of social comparison and public scrutiny online. From that perspective, social media exposure may pose unique struggles for athletes, such as increased pressure from athletic expectations, exposure to constant fan judgment, and heightened anxiety and stress related to their athletic image. Therefore, the four articles in this Research Topic are valuable in that they aim to shed light on the use of social media and its impact on the lives and well-being of athletes.

The study by Zhang et al. examines how passive social media use (e.g., viewing others' profiles without actively interacting) is related to anxiety and subjective well-being in young athletes. Zhang et al. demonstrate that passively scrolling through social media accounts can heighten anxiety and diminish well-being through the negative effects of upward social comparison (e.g., comparing oneself to perceived superiors). In sports culture, where performance and athletic image are constantly evaluated, online content can exacerbate psychological pressure. However, importantly, Zhang et al. introduce the concept of positive psychological capital (i.e., confidence in the ability to achieve goals) as a protective factor against the negative effects of online social comparison. Athletes with higher positive psychological capital were less affected by the emotional and mental consequences of passive scrolling, suggesting individual differences in how athletes interpret and respond to online comparison.

Yan et al. extend this phenomenon to the broader discussion of fitness and appearance ideals on social media. They demonstrate how exposure to fitness ideals on social media is related to females' body esteem, specifically through body surveillance. Additionally, for women with high appearance-contingent self-worth, the effects of exposure to fitness and appearance ideals on social media were especially strong. Using objectification theory, which emphasizes that women often believe that their appearance determines their overall value and competence, Yan et al. argue that viewing and engaging with fitness posts on social media can increase women's body surveillance and ultimately lead to lower body esteem. Although their studies did not specifically involve female athletes, their work sheds light on an important phenomenon that can ultimately lead to discussions about the influence of these posts on female athletes. In sports, athletes may view their bodies as central to their identities. Because athletes often use their bodies to perform and excel in their sports, the way they perceive their physical appearance may be especially relevant to their identity and overall self-esteem. Therefore, further research should continue to investigate these impacts of social media in athletes specifically.

Piepiora et al. transition from discussion around the effects of social media use to specific interventions and treatment modalities aimed at addressing athletes' mental health difficulties. As mentioned above, social media platforms can be seen as a double-edged sword. On the one hand, they offer opportunities for self-promotion and connection with fans and athletic communities, but they also expose athletes to potential scrutiny and other harmful online interactions. Based on psychoanalytic theory, such online interactions may provoke distress that is rooted in early experiences. For these athletes, psychodynamic therapy may be a suitable approach for fostering awareness and resolving conflicts. However, as emphasized by Piepora and colleagues, other treatment modalities (e.g., Cognitive-Behavioral Therapy) may be more appropriate, depending on the presenting concerns. Because athletes are a population with unique characteristics and circumstances, they may require tailored interventions. Importantly, engagement with technology and social media use adds another layer of psychological stress that should also be examined and considered when working with athletes in therapeutic settings.

Şirin et al. extend the current collection by examining how social media platforms are used to express social ideologies within sports communities. Focusing on Turkish football fans' Instagram posts, they discuss how social media platforms serve as tools for collective identity and social responsibility. Their findings reveal that sports fans often use online platforms to express solidarity, commiserate about losses, and reinforce their collective identity. However, they tend to engage less in social issues (e.g., poverty, education, environmental sustainability). This research expands the discussion of social media's role in sports, highlighting how engagement on social media platforms can potentially influence critical social issues and ideologies, beyond sports-related themes.

The current collection sheds light on the role of social media in sports, examining both the potential advantages and drawbacks of online social platforms in the sports world. The rapid growth of these platforms necessitates a thorough examination of their impact on athletes' lives, well-being, and safety. Although engagement on these platforms offers opportunities for positive interactions, community building, and the promotion of socially responsible ideas, it can also lead to negative impacts on athletes' well-being and psychological functioning. Therefore, future research should continue examining the relation between social media engagement and athletes' well-being to better inform athletes, coaches, teams, and mental health professionals about best practices for navigating online environments and mitigating potential risks.
